# Intensified Sampling in Response to a *Salmonella* Heidelberg Outbreak Associated with Multiple Establishments Within a Single Poultry Corporation

**DOI:** 10.1089/fpd.2017.2340

**Published:** 2018-03-01

**Authors:** Alice Green, Stephanie Defibaugh-Chavez, Aphrodite Douris, Danah Vetter, Richard Atkinson, Bonnie Kissler, Allison Khroustalev, Kis Robertson, Yudhbir Sharma, Karen Becker, Uday Dessai, Nisha Antoine, Latasha Allen, Kristin Holt, Laura Gieraltowski, Matthew Wise, Colin Schwensohn

**Affiliations:** ^1^ Office of Public Health Science, Food Safety and Inspection Service, U.S. Department of Agriculture, Minneapolis, Minnesota.; ^2^ Office of Public Health Science, Food Safety and Inspection Service, U.S. Department of Agriculture, Washington, District of Columbia.; ^3^ Office of Public Health Science, Food Safety and Inspection Service, U.S. Department of Agriculture, Athens, Georgia.; ^4^ Office of Public Health Science, Food Safety and Inspection Service, U.S. Department of Agriculture, Springdale, Arkansas.; ^5^ Office of Public Health Science, Food Safety and Inspection Service, U.S. Department of Agriculture, Atlanta, Georgia.; ^6^ Office of Field Operations, Food Safety and Inspection Service, U.S. Department of Agriculture, Denver, Colorado.; ^7^ Office of Field Operations, Food Safety and Inspection Service, U.S. Department of Agriculture, Albany, California.; ^8^ Office of Investigation, Enforcement, and Audit, Food Safety and Inspection Service, U.S. Department of Agriculture, Albany, California.; ^9^ Office of Policy and Program Development, Food Safety and Inspection Service, U.S. Department of Agriculture, Washington, District of Columbia.; ^10^ Office of the Assistant Secretary for Preparedness and Response, Office of Emergency Management, U.S. Department of Health and Human Services, (formerly affiliated with Office of Public Health Science, USDA-FSIS), Washington, District of Columbia.; ^11^ Division of Foodborne, Waterborne, and Environmental Diseases, Centers for Disease Control and Prevention, Atlanta, Georgia.

**Keywords:** antimicrobial resistance in bacteria of animal origin, foodborne disease, food safety, poultry, *Salmonella*

## Abstract

On June 28, 2013, the Food Safety and Inspection Service (FSIS) was notified by the Centers for Disease Control and Prevention (CDC) of an investigation of a multistate cluster of illnesses of *Salmonella enterica* serovar Heidelberg. Since case-patients in the cluster reported consumption of a variety of chicken products, FSIS used a simple likelihood-based approach using traceback information to focus on intensified sampling efforts. This article describes the multiphased product sampling approach taken by FSIS when epidemiologic evidence implicated chicken products from multiple establishments operating under one corporation. The objectives of sampling were to (1) assess process control of chicken slaughter and further processing and (2) determine whether outbreak strains were present in products from these implicated establishments. As part of the sample collection process, data collected by FSIS personnel to characterize product included category (whole chicken and type of chicken parts), brand, organic or conventional product, injection with salt solutions or flavorings, and whether product was skinless or skin-on. From the period September 9, 2013, through October 31, 2014, 3164 samples were taken as part of this effort. *Salmonella* percent positive declined from 19.7% to 5.3% during this timeframe as a result of regulatory and company efforts. The results of intensified sampling for this outbreak investigation informed an FSIS regulatory response and corrective actions taken by the implicated establishments. The company noted that a multihurdle approach to reduce *Salmonella* in products was taken, including on-farm efforts such as environmental testing, depopulation of affected flocks, disinfection of affected houses, vaccination, and use of various interventions within the establishments over the course of several months.

## Introduction

In the United States, the average consumer eats 56 pounds of boneless chicken annually (United States Department of Agriculture, Economic Research Service, 2014). Poultry-related salmonellosis is ranked as the fourth most common pathogen–food combination associated with U.S. foodborne illness, accounting for 215,109 illnesses, 4048 hospitalizations, and 79 deaths annually at an estimated cost of $693 million (Batz *et al.*, 2012). Ten percent of human salmonellosis has been attributed to chicken (Interagency Food Safety Analytics Collaboration (IFSAC) Project, 2015). *Salmonella* Heidelberg is among the top 10 serotypes of *Salmonella* associated with human illnesses (Centers for Disease Control and Prevention, 2013). In addition, it is one of the most invasive serotypes, with an estimated 13% of cases developing systemic infection (Jones *et al.*, 2008).

The U.S. Department of Agriculture, Food Safety and Inspection Service (FSIS) coordinates with the U.S. Centers for Disease Control and Prevention (CDC) and state and local public health partners as described in FSIS Directive 8080.3 (FSIS, 2013a) to investigate illness clusters potentially linked to meat, poultry, or processed egg products. A crucial part of these investigations is the administration of enteric illness questionnaires to case-patients by state and local public health officials.

In the hypothesis-generation phase of an investigation, questionnaires capture a variety of exposures that occurred before illness onset. Once a food source is identified, a focused questionnaire may be used to obtain detailed information on consumption of products of interest, such as brand name and place and date of purchase. Regulatory action in response to foodborne illnesses typically requires a combination of epidemiological, microbiological, and product traceback evidence sufficient to implicate a specific food and production establishment.

On June 28, 2013, FSIS was notified by CDC of a multistate cluster of illnesses of *Salmonella* Heidelberg with a rare pulsed-field gel electrophoresis (PFGE) pattern and a high proportion of ill persons reporting chicken consumption before illness onset (Gieraltowski *et al.*, 2016). CDC later added six additional *Salmonella* Heidelberg PFGE patterns into the case definition for this investigation, one of the largest U.S. foodborne illness investigations of 2013.

This article describes the sampling approach taken by FSIS when epidemiologic evidence implicated chicken products from multiple establishments operating under one corporation. The objectives of sampling were to (1) assess process control of chicken slaughter and further processing (FSIS, 2013c) and (2) determine whether outbreak strains were present in products from implicated establishments.

## Materials and Methods

### Selection of establishments for intensified sampling

FSIS worked with public health officials to request shopper card numbers for case-patients in the outbreak to obtain shopper histories and pursue traceback to producing establishments. Traceback was focused on chicken that was purchased raw and cooked at home, based on food histories reported by case-patients during interviews. A likelihood-based approach using traceback information was used to identify establishments potentially associated with illness. This approach accounted for the number and types of chicken products case-patients reported consuming in the week before illness onset, including establishments determined from case-patient food history and traceback. Rankings were used to prioritize establishments for intensified sampling of raw chicken products available for consumers to purchase. Using this approach, four establishments under the same corporate entity (“Company X”) were selected for initial intensified sampling of chicken parts and whole chickens, focusing on the raw chicken products most frequently mentioned during case-patient interviews. Later in the investigation, additional establishments from the same corporation were included in sampling to rule out their involvement in the ongoing outbreak. As part of the sample collection process, data collected by FSIS personnel included category (whole chicken and type of chicken parts), brand, injection with salt solutions or flavorings, and whether product was skinless or skin-on. Analyses were performed using SAS software version 9.4 (SAS Institute, Cary, NC). Odds ratios and confidence intervals for variables of interest (conventional versus organic, corporate versus retail label, skin-on versus skinless, and injected versus noninjected product) were calculated by chi-square tests of independence and proc logistic maximum likelihood estimates. The intensified sampling for this investigation was performed in phases, where the frequency, types of samples, and/or the establishments that were sampled during each time period were informed by ongoing findings from the investigation.

### Sample collection methods and frequency

The sampling program was divided into six phases (Table 1). FSIS inspection personnel collected samples on up to 5 production days each week. Eligible raw chicken products included whole chicken, tenderloins or strips, and various parts (breasts, thighs, or drumsticks). Similar to the FSIS 2012 Raw Chicken Parts Baseline Study (FSIS, 2012), a minimum of 4 pounds of selected product was collected in final packaging when available. To more closely replicate potential consumer *Salmonella* exposures, the products were shipped to one of the FSIS Field Service Laboratories (Eastern Laboratory, Athens, GA; Midwestern Laboratory, St. Louis, MO; or Western Laboratory, Alameda, CA) where samples were rinsed in accordance with FSIS chicken carcass or chicken parts sampling procedures: 400 mL of buffered peptone water was used to rinse either a whole chicken carcass or 4 pounds of raw chicken parts. A 30 mL test portion of the rinsate was analyzed for *Salmonella* according to the FSIS Microbiology Laboratory Guidebook (MLG), Chapter 4 (FSIS, 2014b). Environmental swabbing was conducted for a brief period at selected establishments to assess the effectiveness of sanitation measures between production days. During this same time period, due to ongoing illnesses and hypotheses regarding exposure from handling of retail-ready raw product packages, exterior packaging from a subset of the products was swabbed using a method similar to food-contact surface samples, as per FSIS Directive 10,300.1 (FSIS, 2013c) Section VII, A, 12, and tested for the presence of *Salmonella* before the parts being rinsed for analysis.

**Table 1.** Sampling Plan by Phase and Establishment

*Date range*	*Establishment(s)*	*Chicken product types sampled (total/day)*	*Other sample types collected (total/day)*
Phase I: 9/9/13–9/27/13 (3 weeks)	A, B, C, D	Chicken parts (5/day), whole/rotisserie chicken (3/day), chicken tenderloins/strips (2/day)	None
Phase II: 10/15/13–12/13/13 (9 weeks)	A, B, C, E, F	Chicken parts (4/day), whole/rotisserie chicken (2/day), chicken tenderloins/strips (1/day)	None
Phase III: 12/16/13–1/13/14 (4 weeks)	A, B, C	Chicken parts (3/day), whole/rotisserie chicken (1/day), chicken tenderloins/strips (1/day)	Preoperational environmental samples (4/day); Product surface swab (1/day)
Phase IV: 1/14/14–2/7/2014 (7 weeks)	A, B, C	Chicken parts (3/day), whole/rotisserie chicken (1/day), chicken tenderloins/strips (1/day)	None
Phase V: 3/3/14–8/8/14 (23 weeks)	A, B, C	Chicken parts (1/day)	None
Phase VI: 8/11/14–10/31/2014 (12 weeks)	C	Chicken parts (1/day)	None

For establishments that did not produce tenderloins and those that did not produce whole carcasses, chicken parts were substituted.

*Salmonella* isolates were further characterized by the FSIS Eastern Laboratory using PFGE (Centers for Disease Control and Prevention, 2015), molecular serotyping (FSIS MLG 4 Appendix 1.03), and antimicrobial susceptibility testing (AST) following National Antimicrobial Resistance Monitoring System (NARMS) protocols (Food and Drug Administration, 2015). When intensified sampling first began, the *Salmonella* serogroup was determined for three isolates selected from each sample. All serogroup B isolates were further characterized by molecular serotyping, PFGE, and AST. When more than one serogroup was identified in one sample, all serogroup B isolates were further characterized as described, and one isolate from each of the other identified serogroups was further characterized. Due to the detection of a high number of outbreak strains, after the first 3 weeks of sampling, only a single confirmed *Salmonella* isolate from each sample was further characterized. When there were multiple isolates from the same sample with the same serotype and PFGE pattern, the isolate with resistance to the greatest number of antimicrobial classes was reported.

## Results

By late August 2013, chicken consumed by multiple case-patients in the outbreak was primarily traced back to three establishments from the same corporation. Product sharing among the three establishments was complex, including commingling of parts from different lots of chicken. A diversity of products and production dates was identified through traceback efforts. On September 9, 2013, FSIS initiated intensified sampling in response to the ongoing outbreak of *Salmonella* Heidelberg associated with raw chicken products.

After the first phase of intensified sampling, FSIS identified three establishments within the corporate umbrella that were implicated in the ongoing outbreak. A Public Health Alert and Notice of Intended Enforcement action were issued by FSIS on October 7, 2013 (FSIS, 2013b). In total, 3164 product and environmental samples were analyzed from six different establishments, which were all part of the same corporation. Of these, 2737 samples were taken at three California establishments implicated in the outbreak, including 163 environmental samples and 90 external package swab samples. Another 427 samples were taken at three establishments in Washington and Louisiana.

Across all phases of sampling, 334 samples were positive for *Salmonella*, and 361 *Salmonella* isolates were found in those positive samples. Thirteen different serotypes were identified. Although there was some diversity in serotypes of *Salmonella* seen during the testing, 263 (72.9%) of the isolates were *Salmonella* Heidelberg (Table 2). The second most frequently detected serotype was *Salmonella* Montevideo (12.2%), followed by *Salmonella* Enteritidis (4.2%). All *Salmonella* isolates were analyzed for antimicrobial resistance (AMR), 26.9% were pansusceptible and 48.2% were resistant to three or more Clinical and Laboratory Standards Institute classes of antibiotic compounds (Food and Drug Administration, 2015). As noted, *Salmonella* Heidelberg was the predominant serotype found during intensified sampling from these establishments. In contrast, *Salmonella* Kentucky is the most common serotype isolated from routine FSIS chicken carcass verification testing, at 61% (*n* = 197/325) of positive samples and ∼2% (*n* = 197/8816) of all samples analyzed during 2014 (FSIS, 2015a). In 2014, *Salmonella* Heidelberg was the fifth most common serotype identified in routine FSIS chicken carcass verification testing, at about 3% (*n* = 11/325) of positive samples and about 0.1% (*n* = 11/8816) of all samples analyzed.

**Table 2.** *Salmonella* Serotypes Detected During Intensified Testing and an Overview of Antimicrobial Resistance Findings

Salmonella *Serotype*	*Number of isolates (% of total isolates)*	*Number of pansusceptible isolates (% of serotype total)*	*Number of isolates with resistance ≥3 CLSI classes (% of serotype total)*
*Salmonella* Braenderup	5 (1.4)	5 (100)	0
*Salmonella* Enteritidis	15 (4.2)	14 (93.3)	0
*Salmonella* Hadar	3 (0.8)	0 (0.0)	0
*Salmonella* Heidelberg	263 (72.9)	22 (8.4)	169 (64.3)
*Salmonella* Infantis	1 (0.2)	1 (100.0)	0
*Salmonella* Kentucky	3 (0.8)	0 (0.0)	1 (33.3)
*Salmonella* Montevideo	44 (12.2)	42 (95.4)	1 (2.3)
*Salmonella* Ohio	3 (0.8)	2 (66.6)	0
*Salmonella* Oranienburg	1 (0.2)	1 (100)	0
*Salmonella* Schwarzengrund	1 (0.2)	0	0
*Salmonella* Senftenberg	1 (0.2)	1 (100)	0
*Salmonella* Typhimurium	9 (2.5)	6 (66.6)	0
*S.* I 4,[5],12:i:—	12 (3.3)	3 (25.0)	3 (25.0)
Total	361 (100.0)	97 (26.9)	174 (48.2)

**FIG. 1.**
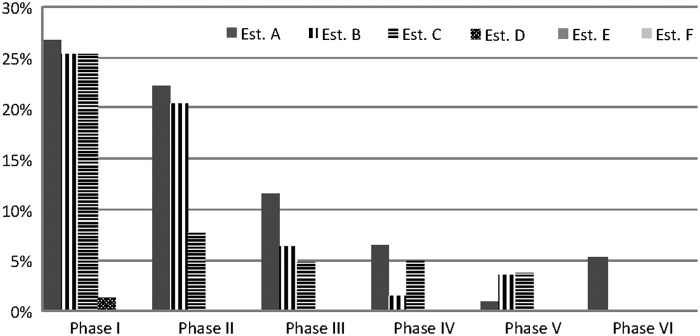
Frequency of *Salmonella* isolation during intensified testing, according to the phase and the analyzed establishment.

From phases I through VI, the percentage of samples positive for *Salmonella* declined from 19.7% to 5.3% (Fig. 1). Intensified sampling identified four (JF6X01.0022, JF6X01.0041, JF6X01.0045, and JF6X01.0258) of the seven outbreak patterns at all three of the California establishments; an additional two (JF6X01.0122 and JF6X01.0326) outbreak patterns were found at two of the three facilities. Pattern JF6X01.0672, a rare PFGE pattern, has never been identified in any FSIS sampling program, including this intensified sampling. It was not until phase IV that the mean percentage of *Salmonella* isolates classified as outbreak strains began to decline (Table 3). During phase V, FSIS determined that a reduced frequency of testing was appropriate to supplement in-plant establishment testing data for continued evaluation of improvements at these establishments. For phase VI, FSIS discontinued intensified sampling at two of the three establishments because both FSIS and establishment sampling results indicated consistent control of *Salmonella*. On October 31, 2014, intensified sampling concluded at the last establishment, after analysis of both FSIS and establishment sampling data supported consistent control of *Salmonella.*

**Table 3.** Proportion of *Salmonella* Positives Identified in Intensified Testing That Were Outbreak Strains, by Establishment and Phase

*Establishment*	*A*	*B*	*C*	*D*	*E*	*F*	*Mean*
Phase I, *N* (% outbreak strains)	29/40 (72.5)	22/38 (56.4)	22/38 (57.9)	1/2 (50.0)			74/118 (62.7)
Phase II, *N* (% outbreak strains)	47/62 (75.8)	40/61 (65.6)	19/23 (82.6)				106/145 (72.6)
Phase III, *N* (% outbreak strains)	15/16 (93.8)	10/11 (90.9)	8/8 (100.0)				33/35 (94.3)
Phase IV, *N* (% outbreak strains)	8/10 (80)	3/3 (100)	9/10 (90)				20/23 (87.0)
Phase V, *N* (% outbreak strains)	2/2 (100.0)	2/4 (50.0)	3/4 (75.0)				7/10 (70.0)
Phase VI, *N* (% outbreak strains)	0/3 (0.0)	—	—				0/3 (0.0)
Total	101	77	61	1			240/334 (71.9)

All the tested preoperational environmental samples (*n* = 163) or external product package swabs (*n* = 90) were negative for *Salmonella* (Table 4). The raw chicken contents of nine of the swabbed packages (10%) were positive for *Salmonella*, and all isolates were outbreak strains. Of 117 organic chicken products sampled at the California establishments, 30.8% were positive for *Salmonella*. Of 2367 conventional chicken products sampled at these establishments, 12.5% were positive for *Salmonella*. Organic chicken products were more likely to be positive for *Salmonella* (OR 3.1, 95% CI: 2.1–4.7), but were less likely to contain outbreak strains (OR 0.3, 95% CI: 0.1–0.5) or isolates exhibiting AMR (OR 0.1, 95% CI: 0.1–0.3) than were conventional chicken products. Chicken labeled with the primary corporate name had similar *Salmonella* percent positive (OR 0.9, 95% CI: 0.7–1.2) to retailer-labeled or other brands of chicken produced by Company X (OR 0.9, 95% CI: 0.7–1.2), but a higher percentage of outbreak strains (OR 1.8, 95% CI: 1.1–2.9) and AMR (OR 2.9, 95% CI: 1.7–4.9). Skin-on products were slightly less likely to be positive for *Salmonella* (OR 0.7, 95% CI: 0.6–0.9) than were skinless products, and had a similar likelihood of containing outbreak strains (OR 1.2, 95% CI: 0.7–1.9) and similar AMR (OR 1.1, 95% CI: 0.6–1.7). Injected products were similarly likely to be positive for *Salmonella* compared with noninjected products (OR 0.9, 95% CI: 0.6–1.2), with similar likelihood of containing outbreak strains (OR 1.1, 95% CI: 0.6–2.2) and similar levels of AMR (OR 2.0, 95% CI: 0.9–4.4). Notably, there was no fluoroquinolone resistance identified in *Salmonella* isolates recovered during intensified testing. A single isolate from an organic chicken product sample exhibited resistance to ceftiofur and cefoxitin (third-generation cephalosporins); however, this isolate was not an outbreak strain. Overall resistance to aminoglycosides, beta-lactams, sulfonamides, and tetracycline was commonly identified, with resistance to chloramphenicol less commonly detected.

**Table 4.** Limited Descriptive Findings for Chicken Products from California Establishments During Intensified Testing, Including Percent of Samples Positive for *Salmonella*, Percentage of Outbreak Strains, and Antimicrobial Resistance Results

*Product class*	Salmonella *positive/total analyzed (%)*	*Odds ratio, 95% CI for* Salmonella *percent positive*	*No. of outbreak strains (% total* Salmonella *isolates)*	*Odds ratio, 95% CI for percent outbreak strains*	*No. of pan-susceptible isolates (% total* Salmonella *isolates)*	*No. of isolates with resistance ≥3 CLSI classes (% total* Salmonella *isolates)*
Organic	36/117 (30.8)	3.1, 2.1–4.7	16/36 (44.4)	0.3, 0.1–0.5	25/36 (69.4)	5/36 (13.9)
Conventional (nonorganic)	296/2367 (12.5)		223/296 (75.3)		62/296 (20.9)	159/296 (53.7)
Environmental samples	0/163 (0.0)		—		—	—
Package swab samples	0/90 (0.0)					
Sold under Company A brand label	170/1395 (12.2)	0.9, 0.7–1.2	132/170 (77.6)	1.8, 1.1–2.9	28/170 (16.5)	94/170 (55.3)
Sold under other retail or brand label	162/1252 (12.9)		107/162 (66.0)		59/162 (36.4)	70/162 (43.2)
Skin-on product^a^	194/1735 (11.2)	0.7, 0.6–0.9	142/194 (73.2)	1.2, 0.7–1.9	50/194 (25.8)	99/194 (51.0)
No skin^a^	138/909 (15.2)		97/138 (70.3)		37/138 (26.8)	65/138 (47.1)
Injected product (conventional only)^a^	49/428 (11.4)	0.9, 0.6–1.2	36/49 (73.5)	1.1, 0.6–2.2	8/49 (16.3)	32/49 (65.3)
Non-injected product (conventional only)^a^	283/2216 (12.8)		203/283 (71.7)		79/283 (27.9)	132/283 (46.6)

^a^ Data on skin and flavor injection not collected for three samples.

As a result of additional analysis performed during phase I, 24 samples were found to contain isolates with up to three different serotypes, PFGE patterns, and/or AMR profiles. Of these, 10 samples yielded one outbreak strain and one non-outbreak strain, 2 samples yielded two outbreak strains, and 1 sample yielded two outbreak strains and one non-outbreak strain.

## Discussion

In this investigation, traceback evidence implicated multiple brands and different types of chicken products, as well as multiple establishments across a broad range of production dates. The intensified sampling approach provided evidence linking multiple establishments operated by a single corporation to a large multistate *Salmonella* Heidelberg outbreak. The high percentage of *Salmonella*-positive samples in chicken carcass and chicken parts in phase I was critical in prompting regulatory actions, including a public health alert (PHA) and notice of intended enforcement (NOIE). These findings also prompted the company's response that led to improved control of *Salmonella* at each of the implicated establishments, as well as an end to illnesses associated with this outbreak investigation. In July 2014, in response to the detection of an outbreak strain in a leftover product from a case-patient's household that was indistinguishable from the case-patient's outbreak strain, the implicated company conducted a voluntary product recall of an unspecified quantity of fresh and frozen raw chicken products (FSIS, 2014a).

The epidemiologic and traceback portions of a large outbreak investigation are resource intensive and are constrained by somewhat limited data (Scallan *et al.*, 2011). These limitations, combined with potential delays in reporting and cluster identification, as well as resource limitations at the local, state, and Federal levels, make all multistate foodborne outbreak investigations a challenge. Chicken-related *Salmonella* investigations pose additional challenges for a number of reasons. An estimated 64.9% of the population reports having consumed chicken prepared at home within the prior week (Foodborne Diseases Active Surveillance Network (FoodNet), 2007); therefore, detecting a significant increase in product consumption exposure within the population of ill case-patients, within the early stages of an outbreak might be difficult. In addition, consumers often purchase and consume multiple brands of chicken, adding to the challenge of case-patient recall and traceback.

Another challenge to narrowing the focus on specific brands of poultry is posed by the fact that large corporations may produce multiple brands and some of their products are sold with retailer-specific brand names. The finding of outbreak strains in chicken with non-Brand X labels that were produced by Company X likely contributed to the complexity of this investigation. Early traceback efforts can be helpful in identifying a common source of contamination when different brand names are associated with one product (Smith *et al.*, 2015). Intensified sampling efforts may also assist in addressing questions regarding common sources of contamination, as with this investigation. In addition, during current outbreak investigations, whole genome sequencing is being utilized when available to narrow sources of poultry-associated illnesses (Crowe *et al.*, 2017).

FSIS does not typically consider *Salmonella* an adulterant in raw poultry products; however, if these products are determined to be injurious to human health, such as in association with an illness outbreak, FSIS may consider the products adulterated. During this outbreak, a recall was initiated when leftover product with identifying package information was tested and found to contain an outbreak strain indistinguishable from that of the case-patient who consumed it in the week before illness onset. As documented in phase I of FSIS intensified sampling, a package of raw chicken purchased by a consumer may carry more than one strain of *Salmonella*. Due to standard protocols and added costs, routine clinical and food testing do not typically involve recovery of multiple isolates from samples; so matching leftover product isolates with case-patient isolates can present a challenge.

There is some evidence that multidrug-resistant strains of *Salmonella* may be associated with increased virulence (Varma *et al.*, 2005; Gebreyes *et al.*, 2009; Gokulan *et al.*, 2013), and the 38% hospitalization rate for this outbreak was high compared to the typical hospitalization rate for all *Salmonella* (22%) (Jones *et al.*, 2008). A comparison of *Salmonella* percent positive on organic versus conventionally produced chicken indicates that although there was less AMR found in isolates recovered from organically produced chicken products, these samples had a higher *Salmonella* percent positive overall. It should be noted that specific production practices such as organic production were not used as selection criteria when samples were collected. The results of organic versus conventional livestock production are an area of ongoing and important research interest.

Although both FSIS and company analysis demonstrated a steady decline in *Salmonella* percent positive on tested chicken as improvements to the food safety systems in these establishments continued, an observed increase in percentage of outbreak strains identified during phases II through IV may help explain the extended duration of this outbreak. The lack of *Salmonella* in preoperational environmental samples suggests that a cleanup between production days was sufficient to prevent cross-contamination from day-to-day operations. However, it is possible that biofilm formation on equipment surfaces or practices of comingling processed parts during production may have played a role in cross-contamination throughout individual production days (Wang *et al.*, 2013; De Oliveira *et al.*, 2014).

The company's stated goal in response to regulatory action was to produce chicken parts with less than 5% of samples positive for *Salmonella*. This goal was less than the national industry prevalence for these product types (26%), which was the basis FSIS used for evaluating process control for chicken parts in these establishments (FSIS, 2012). The company noted that a multihurdle approach to reduce *Salmonella* in products was taken, including on-farm efforts such as environmental testing, depopulation of affected flocks, disinfection of affected houses, vaccination, and use of various interventions within the establishments over the course of several months.

The actions taken by FSIS during and after this outbreak will help reduce the *Salmonella* burden across the poultry industry. In January 2015, FSIS published a Federal Register Notice (FRN) proposing new pathogen reduction performance standards for *Salmonella* and *Campylobacter* in raw chicken parts and comminuted chicken and turkey products (FSIS, 2015b). The FRN also announced FSIS' intent to use routine sampling throughout the year at all eligible establishments rather than infrequently sampling a subset of eligible establishments on consecutive days for a discrete sample set to assess whether establishments' processes are effectively addressing *Salmonella* on poultry carcasses, chicken parts, and comminuted chicken and turkey products. In February 2016, FSIS published final performance standards for these products. FSIS is committed to encouraging reduction of *Salmonella* and *Campylobacter* in poultry products with the goal of foodborne illness prevention.
